# Self-Construals, Anger Regulation, and Life Satisfaction in the United States and Japan

**DOI:** 10.3389/fpsyg.2016.00768

**Published:** 2016-05-31

**Authors:** Satoshi Akutsu, Ayano Yamaguchi, Min-Sun Kim, Atsushi Oshio

**Affiliations:** ^1^Hitotsubashi UniversityTokyo, Japan; ^2^National Graduate Institute for Policy StudiesTokyo, Japan; ^3^University of Hawaii, ManoaHI, USA; ^4^Waseda UniversityTokyo, Japan

**Keywords:** culture, independence, interdependence, anger regulation, life satisfaction

## Abstract

Previous studies have reported evidence that indicates differences between Western and East Asian cultures in anger regulation and its psychological consequences. However, many of these studies have focused on a specific anger regulation strategy and its relation with a psychological consequence. Here, we developed an integrated model that can comprehensively examine three different anger regulation strategies (anger suppression, expression, and control), independent and interdependent self-construals as the psychological antecedent, and life satisfaction as the psychological consequence. We estimated the model using large samples of American and Japanese adults to examine the associations between the two self-construals, three anger regulation strategies, and life satisfaction. We compared the difference in the patterns of relationships among the key constructs between the American and Japanese samples. The results confirmed previously suggested cultural differences while also discovering new culturally different paths. The results generally suggest that individual-level self-construals matter more when anger is a culturally condoned emotion (vs. condemned). The implications and limitations of the integrated model are discussed.

## Introduction

Research into emotion regulation has increased dramatically in the past couple of decades (Gross, 2014). Initial research focused more on individual differences in the use of different emotion regulation strategies and showed that effective regulation of key emotions, such as anger and pride, in everyday life mattered to one’s life satisfaction (Gross, 2007). More recently, the focus has been extended to cultural differences in the use of emotion regulation strategies as well as to how their relationships with the key antecedents and consequences systematically differ across cultures (Mauss and Butler, 2010; Mesquita et al., 2014).

In recent years, there has been an increase in studies examining how one’s culturally motivated goals influence emotion regulation strategy as an antecedent (e.g., Park et al., 2013) and how a specific emotion regulation strategy affects one’s well-being as a consequence (e.g., Kitayama et al., 2015). However, there is a limited amount of studies that comprehensively explore the relationships between emotion regulation strategy and its antecedents (or moderators) and consequences (Butler et al., 2007).

This paper closes a significant gap in the literature by proposing an integrated model that can systematically examine three different approaches of anger regulation (i.e., anger suppression, anger expression, and anger control), independent and interdependent self-construals as the psychological antecedent, and life satisfaction as the psychological consequence. We estimated the model using large representative samples of American and Japanese adults to examine the associations between the two self-construals, three anger regulation approaches, and life satisfaction, and to compare the systematic patterns between the USA and Japan.

### Anger Regulation as a Mediator of Cultural Self-Construals on Life Satisfaction

Independent self-construal refers to a self-conception that values autonomy and independence of individuals, whereas interdependent self-construal refers to that values connectedness and interdependence (e.g., Markus and Kitayama, 1991). Theoretically, each individual possesses both while the balance differs across situations within an individual, across individuals within a culture, and across cultures on average (Benet-Martinez et al., 2002; Oyserman et al., 2002). Life satisfaction refers to a judgment process, in which individuals assess the quality of their lives based on their own unique set of criteria (Shin and Johnson, 1978; Pavot and Diener, 1993). It is regarded as a key manifestation of self-construal among other well-being related constructs such as affective well-being (Markus and Kitayama, 1991; Kitayama et al., 2007), and recently, some studies suggested that the effects of self-construals on life satisfaction were mediated by anger regulation. As a specific example, Novin et al. (2014) showed that emotion expression, along with social support provision, mediated the effects of self-construals on life satisfaction both in the USA and Japan. In the following, we argue that anger regulation strategies mediate the effects of self-construals on life satisfaction and that they do differ between the USA and Japan.

Kitayama and Markus (1994) and Kitayama et al. (2004) suggested that self-construals may critically shape the ways in which individuals regulate their emotions. They claimed that emotion regulation strategies may be culturally reinforced to serve either an interdependent or independent goal. Thus, cultural models of being provide a theoretical foundation for examining the cultural differences in emotion regulation. Mesquita et al. (2014) argued that emotions are culturally afforded to the extent that they benefit the central concerns of the culture. Specifically using anger as a concrete example, they argued that one’s anger implies an attitude of not accepting the situation, an assessment that one has a relatively high level of control over other people in the situation (Frijda et al., 1989), and a willingness to influence these other people so that they accommodate one’s own wishes, goals, and values (Stein et al., 1993). Anger is a well-studied emotion, especially in cross-cultural contexts, presumably because it is an emotion that highlights the independent model of the self, which is dominant in Western cultures, including North America. That is, anger marks individual entitlement and sets clear boundaries, and is thus beneficial in achieving the central goals of the American culture of competitive individualism (Boiger et al., 2013a).

According to Kitayama et al. (2004), the culture may shape emotional experience via collective and personal processes. In contrast to the independent model of the self, Markus and Kitayama (1991) argued that the interdependent model of the self, which is dominant in East Asian cultures, emphasizes connectedness with other people; the self becomes meaningful only in the larger context of social relationships. Thus, although each of the self-construals would function within an individual similarly across cultures, the directions of the primary concern in social contexts could be opposite between the two types of culture.

Specifically, Boiger et al. (2013b) showed that anger was thought to be a culturally condoned emotion in the USA but a culturally condemned emotion in Japan, and thus the perceived frequency of anger situations (i.e., a situation that elicits anger) was higher in the USA than in Japan. If anger is a culturally condoned emotion, whether or not to regulate it is likely to be up to individual values-based preference in the context of USA cultural group. In contrast, if anger is a culturally condemned emotion, it is likely to be socially or structurally regulated so that it will not be expressed in order to best maintain social harmony in the context of Japanese culture. As a result, individuals may not have much freedom to engage in particular anger regulation strategies based on their own level of independent or interdependent self-construal. To support this view of distinguishing between different levels of anger regulation, Mesquita et al. (2014) argued that culture should be defined both at the level of the individual and at the level of the social environment and showed that the broadly defined culture plays an important role in emotion regulation. Similarly, De Leersnyder et al. (2013) identified relational co-regulation and socially structured cultural norms, in addition to individual tendencies, as sources of emotion regulation in a cultural context.

Following Spielberger (1996), in this study, we consider three strategies of anger regulation: anger-in, anger-out, and anger control. Each of the three anger regulation strategies is reviewed below in terms of its relationship with self-construals and life satisfaction, in both independent and interdependent cultural contexts, in order to further explore hypotheses.

### Anger-in/Anger Suppression

Anger-in or anger suppression, is defined as the tendency to turn one’s anger inward, implying anger regulation by suppression (Greenglass, 1996). It is also regarded as the frequency with which angry feelings are experienced but not expressed (Spielberger, 1996). It is related to conflict avoidance, guilt, irritability, decreased life satisfaction, rumination, and depressive symptoms (Kopper and Epperson, 1996; Bridewell and Chang, 1997; Gross and John, 2003; Martin and Dahlen, 2007; McRae et al., 2011; English et al., 2012). Those who can suppress their angry feelings also have a stronger perception of inadequate social support (Palfai and Hart, 1997). Therefore, anger suppression may be related to mental health problems that lead to lower levels of life satisfaction.

Eng et al. (2013, Unpublished) showed that cultural self-construals mediate the cultural differences in the suppression of some emotions. Suppressing emotion basically goes against mainstream American beliefs that put a high value on authenticity (English et al., 2012) and view high arousal emotions as ideal (Tsai et al., 2006). Moreover, since anger is a condoned emotion in the independence dominant American culture, in which it is culturally appropriate to express anger to communicate one’s independence to a larger extent (Boiger et al., 2013a,b), a more highly independent self-construal would inhibit the use of anger suppression to maintain the level of life satisfaction. For the same reason, even those who are high in interdependent self-construal would choose anger control rather than anger suppression to maintain relational harmony. As discussed above, although cross-ethnicity studies in the USA suggest a positive impact of interdependence and a negative impact of independence on anger suppression (e.g., Butler et al., 2007), in Japan where anger is condemned and not expressing negative emotion is a rather strict social norm, we argue that, regardless of their self-construal, people in general are likely to suppress anger in social situations where relational harmony matters and that the impact of self-construals are small.

Although, previous studies suggest the negative impact of anger suppression on life satisfaction in Japan (e.g., Eng et al., 2013, Unpublished), to our knowledge, there is no evidence or theoretical insights that can be used to predict cross-cultural differences in the degree of the negative impact of anger suppression on life satisfaction. In Japan where anger is condemned, people would be used to suppressing anger and thus it would have less of a negative impact on life satisfaction, while people would feel more stress if they are forcibly restrained to keep anger inside by structural norms no matter what their individual values are. Thus, we will make no *a priori* prediction regarding the cultural *differences* in the degree of negative impact of anger suppression on life satisfaction.

### Anger-out/Anger Expression

Anger-out, or anger expression, is an emotion regulation strategy of expressing one’s anger outwardly, usually directing the response at the target of one’s anger (Spielberger, 1996). People in the independent American culture are more likely to express anger freely, or are even encouraged to do so, when they feel frustration and anger to show culturally sanctioned independence (Mesquita et al., 2014). Therefore, independent self-construal is expected to positively affect anger expression in the USA. Even in the independent USA culture, however, interdependent self-construal is expected to negatively affect anger expression in order to avoid disrupting relational harmony (Butler et al., 2007; Novin et al., 2014). The previous literature suggests mixed effects of anger expression on life satisfaction in the USA, it is difficult to make a clear prediction. See, for example, Mesquita et al. (2014) for positive effects and Bridewell and Chang (1997) for negative effects.

Evidence from Japan seems more complicated regarding anger expression. On the one hand, anger expression due to frustration is condemned in Japan in order to better maintain culturally valued social harmony (Boiger et al., 2013b). Therefore, higher interdependent self-construal is likely to negatively affect anger expression in Japan, while the positive impact of independent self-construal may be rather limited to expressing anger that reflects frustrated experience due to the strong cultural norm of interdependence (Kitayama et al., 2015).

On the other hand, in addition to anger expression reflecting frustration, previous studies suggest that there is another motivation of anger expression, which is to display dominance, privilege, and authority (Hurd and Enquist, 2001; Tiedens, 2001). While both the frustration and dominance facets of anger were universally recognized, Park et al. (2013) demonstrated that the dominance facet of anger expression was more prominent than the other frustration-reflecting facets of anger expression in Japan whereas the opposite was the case in the USA. Specifically, Park et al. (2013) demonstrated that those with lower subjective socioeconomic status were more likely to express their anger among USA samples whereas those with higher objective socioeconomic status were more likely to express their anger among Japanese samples, and that the effects in the USA were mediated by frustration while the effects in Japan were mediated by decision authority. Kitayama et al. (2015) extended the findings of Park et al. (2013) and further demonstrated that expression of the dominance facet of anger was positively associated with various health outcomes in Japan whereas the expression of the frustration facet of anger was negatively associated with various health outcomes in the USA.

While such previous research has demonstrated that independent self-construal positively affects the frustration facet of anger expression in the USA, to our knowledge no empirical evidence is available regarding which of the two self-construals, if any, affects the dominance facet of anger expression in interdependent dominant cultures. It is anticipated, however, that higher independent self-construal will positively affect the dominance facet of anger expression because it is based on an independence-oriented motivation to differentiate oneself from others by affirming one’s dominant and privileged status.

Due to this multi-faceted nature of anger expression in Japan, its impact on life satisfaction is not clear. Thus, we will explore the data without *a priori* prediction regarding this. Similarly, it is hard to make *a priori* predictions regarding the cultural *differences* in the degree of the association between anger expression and both its antecedents as well as its consequence.

### Anger Control

Anger control refers to an emotion regulation strategy of controlling anger internally, staying calm, and not expressing it outwardly (Spielberger, 1996). While it is similar to anger suppression, in that anger is not expressed externally, anger control would involve some kind of cognitive processes such as reappraisal (Gross, 1998, 2014). Reappraisal refers to the ability to monitor and prevent the experience or expression of anger by cognitively changing the feeling of anger (Bridewell and Chang, 1997). Thus, it would encourage adaptive functioning and help to promote and recognize the utility of efforts to adjust the intensity or duration of the emotion. It may not result in eliminating the emotion or escaping it altogether (Gratz and Tull, 2010; McRae et al., 2011; English et al., 2012; Roberton et al., 2014).

Using samples of young American adults, Bridewell and Chang (1997) demonstrated that (1) anger suppression, followed by a lack of anger control, played a key role in predicting depressive and anxious symptoms; (2) anger expression, along with gender, played a key role in predicting depressive symptoms but not anxious symptoms; and (3) anger expression, followed by a lack of anger control and anger suppression, played a key role in predicting hostility. In the USA culture, high interdependent self-construal would be strongly associated with high anger control as the best solution for achieving the individually held value of interdependence (i.e., maintaining relational harmony) while minimizing culturally salient negative consequences of anger suppression (e.g., high anxiety and depressive symptoms). However, although saving relational harmony would promote life satisfaction while avoiding the negative impact of suppressing anger, compromising one’s independence would lower life satisfaction of people in the independence dominant American culture. Because these mixed effects would cancel each other out, it is expected that the impact of anger control on life satisfaction in the USA may not be substantially large while it is hard to predict its direction *a priori*. Contrary to interdependence, independence would inhibit anger control as it inhibits anger suppression. However, due to its limited negative consequence, if any, the negative impact of independence would be rather small.

As discussed, in Japan where anger is condemned and not expressing negative emotion is a social norm, regardless of their self-construal, people in general are likely to suppress anger in social situations where relational harmony matters. Moreover, there are no previous studies, to our knowledge, that indicate a systematic influence of either independence or interdependence on the choice between anger suppression and anger control. Thus, we do not predict any substantial impact of both self-construals on anger control in Japan. No matter who chooses it, however, anger control is predicted to positively relate to life satisfaction in the interdependent dominant Japanese culture where compromising showing one’s independence may not be as valued as maintaining relational harmony.

### Model

**FIGURE 1:**
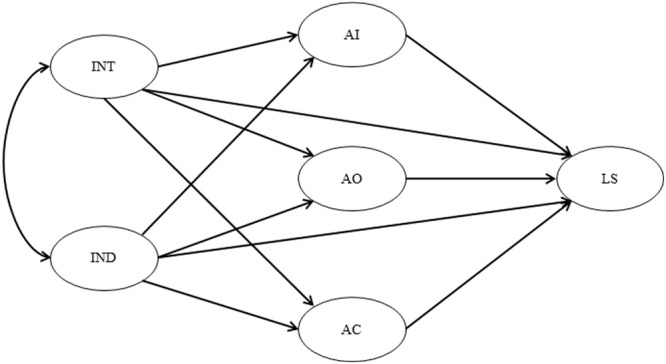
**The conceptual model.** IND, independent self-construal; INT, independent self-construal AI, anger-in; AO, anger-out; AC, anger-control; LS, satisfaction with life scale.

A graphical presentation of the conceptual model that links the aforementioned factors is provided in **Figure 1**.

In summary, in the USA culture where anger is condoned, it is expected that independent self-construal will be negatively related to anger suppression but positively related to anger expression, and will have a limited negative impact on anger control, if any. In contrast, it is expected that interdependent self-construal will be negatively related to anger expression but positively related to anger control, and will have only a small negative impact on anger suppression, if any. Regarding the outcome, it is expected that anger suppression will be negatively related to life satisfaction. However, no clear prediction regarding the impact of anger expression and anger control on life satisfaction will be made because each of them appears to have opposing effects. While the impact of anger control on life satisfaction is not expected to be large anyway, it is hard to predict its direction *a priori*.

In the Japanese culture where anger is condemned, it is expected that independent self-construal will be positively related to anger expression, yet will have rather a small positive impact, if any, on anger suppression or anger control. In contrast, interdependent self-construal will be negatively related to anger expression, yet will have rather a small negative impact on anger suppression or anger control. Anger suppression is predicted to be negatively related to life satisfaction, whereas anger control is predicted to be positively related to life satisfaction. We made no *a priori* prediction regarding the impact of anger expression on life satisfaction due to its mixed impact.

Regarding the cultural difference in the degree of association among factors, we predicted that the negative impact of independence on anger suppression and the positive impact of interdependence on anger control will be larger in the USA and that the positive impact of anger control on life satisfaction will be larger in Japan. We empirically explored the cultural differences of other associations among factors without making clear predictions.

Based on the empirical evidence from previous cross-cultural studies using similar factors (e.g., Park et al., 2013; Novin et al., 2014; Kitayama et al., 2015), we assumed and established the measurement equivalence of each construct (i.e., independent/interdependent self-construal, anger-in, anger-out, anger control, and life satisfaction) between the two national samples. We anticipated that the cultural moderation effects demonstrated in the previous studies would be reflected in different patterns of relationship between emotion regulation, its antecedents, and its consequence in the American and Japanese samples.

## Materials and Methods

### Participants

We used a subset of the Midlife Development in the United States (MIDUS) survey and the corresponding Midlife Development in Japan (MIDJA) survey conducted in 2008 to estimate our model (for a more comprehensive review of the MIDUS and MIDJA, please refer, for example, to Park et al., 2013). For the USA sample, we used the MIDUS Project 4 of the second wave of the MIDUS (i.e., MIDUS II) conducted in 2004. MIDUS II is the longitudinal follow-up data from the MIDUS I that was conducted in 1995 and 1996. The MIDUS II used a subsample of participants (*N* = 1,255) from the original MIDUS study (MIDUS I). The final USA sample consisted of 542 males and 713 females, aged from 35 to 86 years-old (*M* = 57.32, *SD* = 11.5). For the Japanese sample, the MIDJA survey was used (*N* = 1,027). The final Japanese sample consisted of 505 males and 522 females aged 30–79 years-old (*M* = 54.3, *SD* = 14.1). The *t*-test of age between the USA and Japanese cultures was conducted (*t* = 5.64, *df* = 2280, *p* < 0.001, *d* = 0.23). The chi-square test of gender and culture was also performed (χ^2^ = 8.15, *df* = 1, *p* < 0.01, *r* = 0.06). Even though these differences are statistically significant, effect sizes are small. “Japanese respondents completed self-administered questionnaires; the Japanese version was back-translated and adjusted multiple times by native speakers to generate analogous meaning” (Curhan et al., 2014).

**Table 1** shows the sample size, mean, and standard deviation for each variable in the USA and Japan. As described above, it should be noted that MIDUS and MIDJA samples are not truly a national representative sample although they are much more nationally representative than rather limited student samples. Most notably, they are biased toward older adults. It would be a part of the reason for higher mean of interdependence in the USA.

**Table 1:** Mean and standard deviation for the study measures in the USA and Japan.

	USA			Japan		
	*N*	*M*	*SD*	*N*	*M*	*SD*
(1) IND	1248	5.20	0.82	1019	4.66	0.76
(2) INT	1250	5.17	0.66	1021	4.75	0.67
(3) Anger-in	1250	14.65	4.16	1017	14.16	3.67
(4) Anger-out	1251	12.91	3.30	1019	12.17	3.43
(5) Anger control	1252	9.92	2.28	1015	7.95	2.54
(6) Life satisfaction	1249	4.78	1.31	1020	4.07	1.21

*IND, independent self-construal; INT, interdependent self-construal.*

### Measurement Instruments

The Self-Construal Scale (Singelis, 1994) was used to measure interdependent and intdependent self-construals. Responses to the 10 interdependent and 7 independent items were measured on a seven-point scale, ranging from 1 (*strongly disagree*) to 7 (*strongly agree*). The confirmatory factor analysis (CFA) was conducted to examine our proposed model and test the internal consistency of measurement items. As a result, some items were dropped. The remaining items include “It is important for me to maintain harmony or smooth relationships within my group” for interdependence and “Speaking up is not a problem for me” for independence (see **Table 2** for remaining items with path coefficients). Cronbach’s alphas of interdependence were 0.60 (USA) and 0.60 (Japan) and those of independence were 0.63 (US) and 0.62 (Japan).

**Table 2:** Path coefficients of Self-Construal Scale.

	Japan			USA		
	B	*SE*	β	B	*SE*	β
Interdependence: INT
(a) I have respect for the authority figures with whom I interact.	0.84	0.06	0.36	0.84	0.06	0.45
(c) It is important for me to maintain harmony or smooth relationships within my group.	1.00	0.00	0.52	1.00	0.00	0.55
(i) I will sacrifice my self-interest for the benefit of the group I am in.	1.25	0.07	0.58	1.25	0.07	0.58
(j) I should take into consideration others’ advice when making work or family plans.	1.03	0.07	0.49	1.03	0.07	0.54
(m) I will stay in a group if they need me, even when I’m not happy with the group.	0.99	0.07	0.44	0.99	0.07	0.40
(n) If people in my family fail, I feel responsible.	0.74	0.07	0.37	0.74	0.07	0.26
Independence: IND
(b) I’d rather say “NO” directly, than risk being misunderstood.	0.54	0.04	0.55	0.54	0.04	0.37
(d) Speaking up is not a problem for me.	1.00	0.00	1.00	1.00	0.00	0.66
(e) Having a lively imagination is important to me.	0.73	0.04	0.52	0.73	0.04	0.55
(f) I am comfortable with being singled out for praise or reward.	0.59	0.04	0.59	0.59	0.04	0.43
(k) I prefer to be direct and forthright when dealing with people I’ve just met.	0.69	0.04	0.69	0.69	0.04	0.54

Anger regulation was measured using anger-in, anger-out, and anger control subscales of the State-Trait Anger Expression Inventory, each of which refers to the extent to which “one can keep angry feelings inside or can suppress anger or furious feelings,” “one can express feelings of anger, furious feelings, or lose control,” and “one can control anger or furious feelings using physical or verbal expression and communication,” respectively (STAXI; Spielberger, 1996). Responses to the eight anger-in, eight anger-out, and four anger control items were measured on a four-point scale, ranging from 1 (*almost never*) to 4 (*almost always*). The CFA was conducted to examine our proposed model and to test the internal consistency of the measurement items. One item was dropped. Sample items are as follows: in general when I feel angry or furious, I withdraw from people (anger-in); I express my anger (anger-out); and I control my temper (anger control). See **Table 3** for all the remaining items with path coefficients. Cronbach’s alphas of anger-in were 0.82 (USA) and 0.75 (Japan), those of anger-out were 0.77 (USA) and 0.80 (Japan), and those of anger control were 0.83 (USA) and 0.78 (Japan).

**Table 3:** Path coefficients of Spielberger Anger Expression Inventory.

	Japan			USA		
	B	*SE*	β	B	*SE*	β
Anger-in (AI)
(a) I withdraw from people.	0.82	0.06	0.48	0.82	0.06	0.56
(b) I pout or sulk.	1.00	0.00	0.50	1.00	0.00	0.55
(c) I am angrier than I’m willing to admit.	1.36	0.06	0.63	1.36	0.06	0.64
(d) I am secretly critical of others.	1.10	0.06	0.56	1.10	0.06	0.56
(e) I boil inside, but don’t show it.	1.27	0.06	0.48	1.27	0.06	0.63
(f) I harbor grudges.	0.94	0.05	0.51	0.94	0.05	0.52
(g) I keep things in.	1.26	0.07	0.45	1.26	0.07	0.57
(h) I am irritated more than others are aware.	1.64	0.07	0.68	1.64	0.07	0.76
Anger out (AO)
(i) I slam doors.	0.62	0.03	0.47	0.62	0.03	0.53
(j) I say nasty things.	1.00	0.00	0.72	1.00	0.00	0.72
(k) I make sarcastic remarks.	1.00	0.04	0.71	1.00	0.04	0.64
(l) I argue with others.	0.77	0.04	0.45	0.77	0.04	0.55
(m) I lose my temper.	0.91	0.03	0.67	0.91	0.03	0.66
(n) I strike out at whatever infuriates me.	0.65	0.03	0.65	0.65	0.03	0.53
(o) I express my anger.	0.91	0.04	0.66	0.91	0.04	0.52
(p) If someone annoys me I tell them how I feel.	0.57	0.04	0.38	0.57	0.04	0.31
Anger control (AC)
(q) I control my temper.	0.83	0.02	0.68	0.83	0.02	0.78
(r) I keep my cool.	1.00	0.00	0.93	1.00	0.00	0.94
(s) I calm down faster.	0.70	0.02	0.65	0.70	0.02	0.64

Life satisfaction was measured by the Satisfaction with Life Scale (Pavot and Diener, 1993). Four-point scale, ranging from 1 (*not at all important*) to 4 (*extremely important*), was used. The CFA was conducted to examine our proposed model and test the internal consistency of the measurement items. No items was dropped. The remaining five items include “I am satisfied with my life” (see **Table 4**). Cronbach’s alphas of life satisfaction were 0.88 (USA) and 0.90 (Japan).

**Table 4:** Path coefficients of Satisfaction with Life Scale.

	Japan			USA		
	B	*SE*	β	B	*SE*	β
Satisfaction with Life Scale						
(b) In most ways my life is close to my ideal.	1.00	0.00	0.89	1.00	0.00	0.81
(c) The conditions of my life are excellent.	1.05	0.02	0.92	1.05	0.02	0.86
(d) I am satisfied with my life.	1.03	0.02	1.03	0.02	0.02	0.88
(e) So far I have gotten the important things I want in life.	0.85	0.02	0.75	0.85	0.02	0.74
(f) If I could live my life over, I would change among nothing.	0.73	0.03	0.55	0.73	0.03	0.55

## Results

### Preliminary Analyses

Before examining the relationships between the key constructs, we conducted a CFA in the structural equation modeling (SEM) of all of the factors in order to examine the factorial commonalities between the two samples (i.e., the USA and Japan) with equality constraints (i.e., assuming that the covariance among all of the factors). Using AMOS 18 (Arbuckle, 2009), SEM was conducted to examine two models: an unconstrained model, in which no path coefficients were constrained to be equal for both cultures, and a constrained model, in which all path coefficients were constrained to be equal for both cultures. Because the model fit index score with the constrained model with all path coefficients are not significant different (Δχ^2^ = 7.01, *df* = 30, *n.s.*). This can confirm the cross-cultural similarities. There were covariates among all latent variables in both models. As suggested by Raykov et al. (1991), the SEM results were evaluated using two model fit indexes: the comparative fit index (CFI) and the root-mean square-error of approximation (RMSEA). Fit indices of the unconstrained model were as follows: χ^2^ = 4824.77, *df* = 1090, *p* < 0.001, CFI = 0.85, and RMSEA = 0.039; and for the constrained model were as follows: χ^2^ = 5143.19, *df* = 1118, *p* < 0.001, CFI = 0.84, and RMSEA = 0.040. Model fit indices over 0.95 and an RMSEA of 0.06 or less are regarded as indicative of good model fit (Bollen, 1989; Bentler, 1990; Hu and Bentler, 1999). The RMSEA values in this study indicate that both models have a very good fit to the data. Although, the unconstrained model was better than the constrained model in terms of the CFI, there were no substantial differences in fit between the two models; both were a very good fit in terms of RMSEA values. We decided to adopt the constrained model (i.e., with factor equivalence across the two cultural groups) as the baseline for the subsequent analyses, as our main interest was in determining how the associations of the latent variables differ between the two cultures.

**Table 5** shows the correlation matrix of the key variables as well as age and gender. The coefficients were estimated using the constrained model of the CFA, and were all significant at *p* < 0.005, if not *p* < 0.001, except for the correlation between independent self-construal and anger-out in the USA, and the correlations in Japan between independent self-construal and anger-in; interdependent self-construal and both anger-in and anger-out; anger-out and anger control; and anger control and life satisfaction.

**Table 5:** The standardized path coefficients of correlations in the USA and Japan.

	1	2	3	4	5	6
(1) IND USA Japan	–	0.192∗∗ 0.325∗∗	-0.204∗∗ 0.080	0.092∗∗ 0.115∗	0.109∗∗ 0.098∗∗	0.171∗∗ 0.192∗∗
(2) INT USA Japan		–	0.028 -0.089	-0.080∗∗ -0.045	0.169∗∗ 0.087∗∗	0.273∗∗ 0.208∗∗
(3) AI USA Japan			–	0.260∗∗ 0.837∗∗	-0.170∗∗ 0.098	-0.328∗∗ -0.091
(4) AO USA Japan				–	-0.319∗∗ -0.010	-0.175∗∗ -0.029
(5) AC USA Japan					–	0.209∗∗ 0.046
(6) LS USA Japan						–

*Standardized coefficient: (USA, Japan). IND, independent self-construal; INT, interdependent self-construal; AI, anger-in; AO, anger-out; AC, anger control; LS, life satisfaction. Even though there are some significant correlations regarding age and gender, the maximum absolute value of the correlation is less than 0.26. ^∗^*p* < 0.05; ^∗∗^*p* < 0.01.*

### Main Analyses

Our intention was to develop an integrated model with the three types of anger regulation strategies (anger-in, anger-out, anger control) serving as mediators between self-construals and life satisfaction. We conducted a path analysis to test the integrated model as it relates to the effects of self-construals and anger regulation strategies on life satisfaction. A path analysis was conducted in which all path coefficients were constrained to be equal for both cultures. Fit indices of the model were as follows: χ^2^ = 5064.38, *df* = 1118, *p* < 0.001, CFI = 0.84, and RMSEA = 0.039.

**FIGURE 2:**
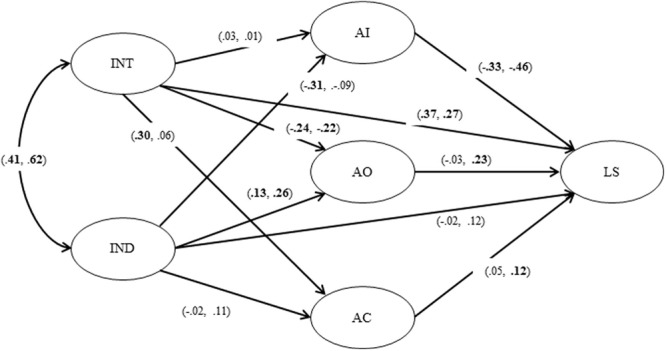
**The model estimation results.** IND, independent self-construal; INT, independent self-construal AI, anger-in; AO, anger-out; AC, anger-control; LS, satisfaction with life scale. The path coefficients are for the US sample. The right side of the path coefficients are for the Japanese sample.

As shown in **Figure 2**, for the USA participants, independent self-construal was negatively related to anger-in, β = -0.31, *p* < 0.01, positively related to anger-out, β = 0.13, *p* < 0.01, and did not directly relate to anger control, β = -0.02, *n.s*. Interdependent self-construal was not related to anger-in, β = 0.03, *n.s.*, but was negatively related to anger-out, β = -0.24, *p* < 0.01, and positively related to anger control, β = 0.30, *p* < 0.01. Life satisfaction was negatively related to anger-in, β = -0.33, *p* < 0.01, but not related to either anger-out, β = -0.03, *n.s.*, or anger control, β = 0.05, *n.s*. Independent self-construal did not directly relate to life satisfaction, β = -0.02, *n.s.*, but interdependent self-construal did directly and positively relate to life satisfaction, β = 0.37, *p* < 0.01. There was covariance between independent self-construal and interdependent self-construal, β = 0.41, *p* < 0.01.

As shown in **Figure 2**, for Japanese participants, independent self-construal was not related to anger-in, β = -0.09, *n.s.*, was positively related to anger-out, β = 0.24, *p* < 0.01, and was not related to anger control, β = 0.11, *n.s*. Interdependent self-construal was not related to anger-in, β = 0.01, *n.s.*, was negatively related to anger-out, β = -0.22, *p* < 0.01, and was not related to anger control, β = 0.06, *n.s*. Life satisfaction was negatively related to anger-in, β = -0.47, *p* < 0.01 but positively related to both anger-out, β = 0.23, *p* < 0.01 and anger control, β = 0.12, *p* < 0.01. Independent self-construal did not directly relate to life satisfaction, β = 0.02, *n.s*, but interdependent self-construal was directly and positively related to life satisfaction, β = 0.27, *p* < 0.01. There was covariance between independent self-construal and interdependent self-construal, β = 0.62, *p* < 0.01.

**FIGURE 3:**
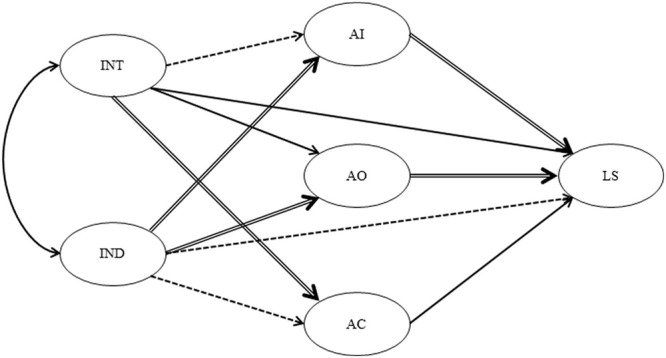
**The USA-Japan difference in the path coefficients.** IND, independent self-construal; conceptual INT, independent self-construal AI, anger-in; AO, anger-out; AC, anger-control; LS, satisfaction with life scale. The double solid lines indicate that significant differences were found in the path coefficients between the US sample and Japanese sample. The single solid lines indicate that no significant differences were found in the path coefficients between the US sample and Japanese sample. The broken lines indicate that no significant differences were found in the path coefficients between the US sample and Japanese sample and that there were no significant effects in both countries.

To address the cultural differences and similarities between the USA and Japanese samples for the same parameters in the proposed model, critical ratio tests were conducted at the level of *p* < 0.05. The results revealed the culturally similar and culturally different processes in the path model (see **Figure 3**).

The culturally different processes between the USA and Japanese samples reflected the effect of independent self-construal on anger-in and anger-out, of interdependent self-construal on anger control, and of anger-in and anger-out on life satisfaction. More specifically, the negative association of independent self-construal with anger-in was significantly larger in the USA sample than in the Japanese sample, whereas its positive association with anger-out was significantly larger in the Japanese sample than in the USA sample. The positive association of interdependent self-construal with anger control was significantly larger in the USA sample than in the Japanese sample. The negative association of anger-in and the positive association of anger-out with life satisfaction were both significantly larger in the Japanese sample than in the USA sample.

Processes that were culturally common (including processes that were not significant in both cultures) were observed in the relationship between independent and interdependent self-construals, the association of independent self-construal with anger control and life satisfaction, and of interdependent self-construal with anger-in, anger-out, and life satisfaction, as well as that of anger control with life satisfaction. More specifically, for both cultures, there was a significantly positive relationship between independent and interdependent self-construals, as well as positive associations of interdependent self-construal and anger control with life satisfaction, whereas the association of interdependent self-construal with anger-out was significantly negative. The association of independent self-construal with anger control and life satisfaction, as well as that of interdependent self-construal with anger-in, was not significant either culture.

## Discussion and Conclusion

While we did not make clear predictions for some associations, especially regarding cultural differences in the association strength, the results, which were based on significance tests, generally supported our proposed path model. As expected for the USA sample regarding the antecedent of anger regulation, independent self-construal negatively related to anger-in and positively related to anger-out. There was no significant relation between independence and anger control, which was no surprise because we did not make a strong prediction.

As expected for the Japanese sample, both anger-in and anger control were not significantly associated with either interdependent or independent self-construal. These results suggest the limited impact of individually held self-construals on regulating anger within oneself in Japan. While anger-in and anger control are negatively correlated, and may be used as exclusive alternatives by the American participants, they are positively correlated in the Japanese participants, and thus may be used as equivalent substitutes.

While both the individual-level difference and the social-level difference are rooted in cultural manifestations, they are not the same and have a different impact. Specifically, their impact on life satisfaction would be different even though they result in the same anger regulation strategy. Although we did not explicitly predict this, because little has been known about how the source of anger regulation interacts with culture and alters the impact of a particular anger regulation strategy on life satisfaction, our results indicated that the cross-culturally common significant negative impact of anger-in was even stronger in the Japanese sample than in the USA sample. One account for this unexpectedly stronger negative association of anger-in for the Japanese sample is that it was due to the social norm’s coercive force making people conform to suppress anger for the sake of social harmony regardless of one’s individually held cultural self-construals, even if one is individually holding very strong independent self-construal.

The positive association of anger control with life satisfaction was significant only in the Japanese sample while there was no significant difference in the strength of the impact between the USA and Japanese samples. Anger control involves cognitively effortful change or reappraisal (Gross and John, 2003; Gross, 2014). Anger control, as specified by Spielberger (1996) and used in this study, comes only after anger is felt. However, given that individually held self-construals have no effect on anger control, anger control by reappraisal could possibly be more automatic, and thus less effortful and stressful for Japanese participants than for American participants (Mauss et al., 2007, 2008).

Consistent with our prediction, anger-out was significantly positively associated with independent self-construal and negatively associated with interdependent self-construal in the Japanese sample, just as in the USA sample. However, it was surprising that the positive association of independent self-construal was significantly stronger in the Japanese sample than in the USA sample. Combined with the result that the *positive* association of anger-out with life satisfaction was significant in the Japanese sample, higher independent self-construal seemed to be associated with more frequent use of “anger-out privilege” in the Japanese sample (Kitayama et al., 2015). For the USA sample, anger-out was not significantly related to life satisfaction, presumably due to the more complex mixed effects of showing independence, venting frustration, destroying relational harmony, and displaying authority (Park et al., 2013; Mesquita et al., 2014; Kitayama et al., 2015). As discussed, whereas anger expression in terms of venting frustration reflects a frustrating experience, and thus links to lower life satisfaction, anger expression in terms of displaying authority reflects one’s dominance over others, and thus links to higher life satisfaction. Moreover, the former is more prominent in the USA whereas the latter is more prominent in Japan (Park et al., 2013; Kitayama et al., 2015). Although this “multi-faceted anger mechanism” was not directly examined, our results are at least consistent with its prediction.

This study explored the overall mechanism of how two types of self-construal (i.e., independent and interdependent) and three strategies of anger regulation (i.e., anger-in, anger-out, and anger control) relate to life satisfaction. It provided evidence that anger regulation partially mediates the relationships between cultural self-construals and life satisfaction. We found a significant relation of interdependent self-construal to anger-out and life satisfaction for both cultures, and to anger control only for the USA. While previous studies identified some antecedents to depression, such as worry and rumination (e.g., Hong, 2007), and suggest its link to life satisfaction, this study was the first to explicitly link anger regulation strategies to life satisfaction. Our results demonstrated systematic cultural differences in the process. Specifically, two out of three pathways from anger regulation to life satisfaction were significantly different and three out of six pathways from self-construal to anger regulation strategies were significantly different between the two cultures.

More importantly, this study increases our theoretical understanding of the factors that feed into life satisfaction across different cultural contexts. In particular, it suggests that future models of emotion regulation strategies and their consequences should pay more attention to individually held self-construals as antecedents because these self-views tend to serve as antecedents of other cultural and individual values and behaviors that ultimately feed into happiness and life satisfaction. The findings also indicate that closely examining the effects of individually held self-construals would provide more insight into other cultural factors at a different level, such as cultural norms and social processes. We believe that our results provide new insights into the differential impacts of individual-level and social/structural-level cultural antecedents of anger regulation strategies on life satisfaction between two cultures.

### Limitations and Directions for Future Research

First, as our model is correlation, one should be cautious in drawing conclusions about the causal relationships between self-construals, anger regulation strategies, and life satisfaction. Further experimental research should be conducted in both cultural groups to explore their causal relationships. Moreover, longitudinal studies would provide insight not only into the direction of the causal relationships but also into the change over time in the same individuals. Second, our data is limited to middle-aged to older adults. With respect to the level of self-construal, for example, Japanese participants reported higher levels of interdependence than independence, whereas American participants did not differ in their level of the two self-construals. Perhaps this age group is more concerned with its in-group members (e.g., family) compared to younger Americans, while at the same time still valuing independence. Without further replication with younger adults, we cannot be certain how much the pathways to life satisfaction uncovered in the present study are valid within a culture. Third, our study samples were limited to participants in the USA and Japan. To investigate wider cultural variations, data must be collected from different locations encompassing diverse cultures. Fourth, the measurement of all of the constructs in this study is by self-report method. Thus, collecting information from various sources and using different measurements in future studies would be helpful. Finally, yet importantly, our study focuses on anger regulation but we believe that examining other emotions will provide synergetic value. For example, examining the regulation of shame, which is condemned in the USA but condoned in Japan (Boiger et al., 2013b), would complementally validate our anger-based results.

In some cultures, including the USA, expression may be fairly undifferentiated (e.g., one feels simply bad, including anger, disgust, being upset, and sad). In contrast, in Japan, expression of anger may be more granular (e.g., anger may be specific to situation A, but not situation B; see Grossmann et al., 2015). Further research is needed to account for cross-cultural and individual variance in emotional complexity measures.

## Conclusion

This study explored the proposed model using large representative samples of American and Japanese adults to examine the associations between the two self-construals, three anger regulation strategies (anger suppression, anger expression, and anger control), and life satisfaction. This study confirmed cultural differences while also discovering new culturally different paths. In the USA sample, there was no significant relation between independence and anger control. By contrast, in Japanese sample, these results indicate the limited impact of individually held self-construals on regulating anger within oneself in Japan. While anger-in and anger control are negatively correlated with independence, and may be used as exclusive alternatives by the American participants, they are positively correlated among the Japanese participants, and thus may be used as equivalent substitutes. Moreover, our findings should have the important implications for health care settings as other contexts. Interventions attempt to reduce or relieve negative affect, although essential in some contexts, may not be universally desired or helpful.

## Author Contributions

SA and AY conceived the study. AO and AY carried out data analysis. SA and AY drafted the manuscript. M-SK provided critical revisions. All authors participated in discussions of the manuscript. They have approved the final version of the manuscript for submission.

## Conflict of Interest Statement

The authors declare that the research was conducted in the absence of any commercial or financial relationships that could be construed as a potential conflict of interest.
